# Supplementary material for: The Relative Positioning of B and T Cell Epitopes Drives Immunodominance
Source: Vaccines (Basel). 2022 Jul 31;10(8):1227. doi: 10.3390/vaccines10081227 (PMC9413633; doi:10.3390/vaccines10081227)
Supplement: Supplementary file 1 [file vaccines-10-01227-s001.zip › Vaccines Supplementary files Revised proofs/vaccines-1784638 supplementary.docx]

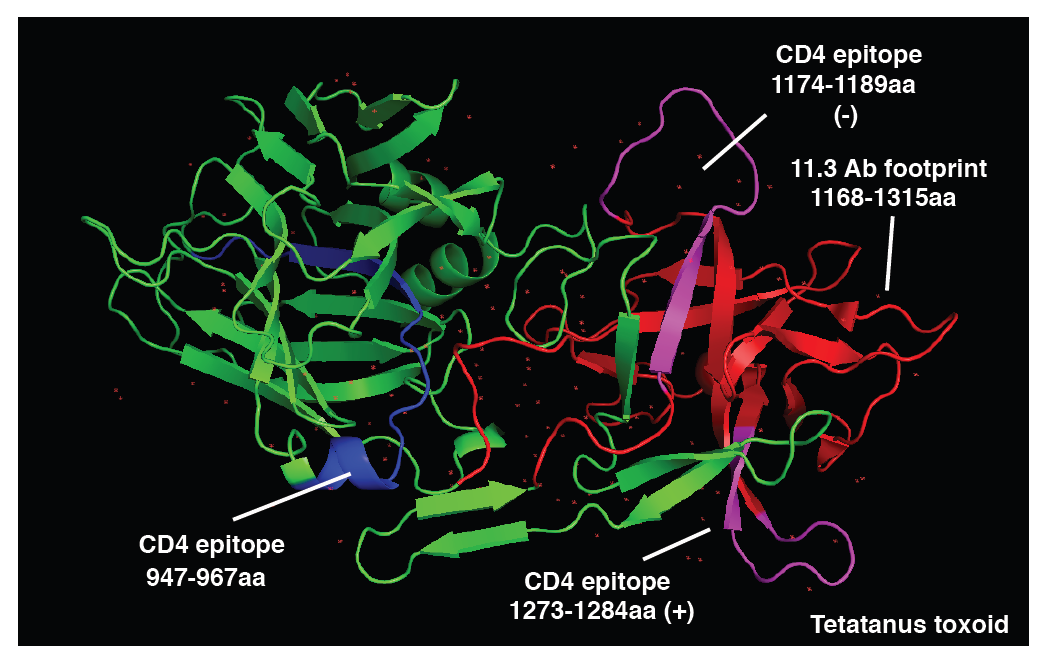


Figure S1. Crystal structure of tetanus toxoid (PDB:1AF9) is shown. Antibody footprint binding region is depicted in red, while CD4 determinants are highlighted in blue.


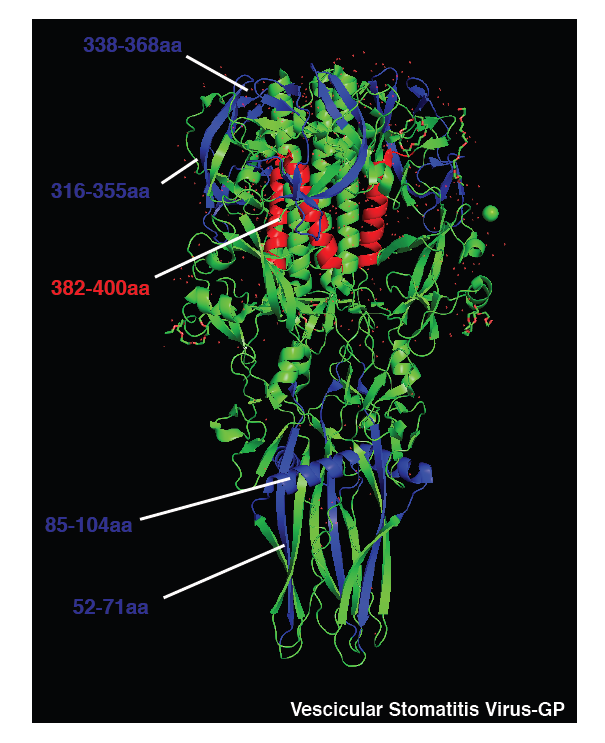


Figure S2. Crystal structure of Vesicular Stomatitis Virus glycoprotein (PDB:512M) is shown. Immunodominant B-cell epitopes are depicted in red, while dominant CD4 determinants are highlighted in blue.


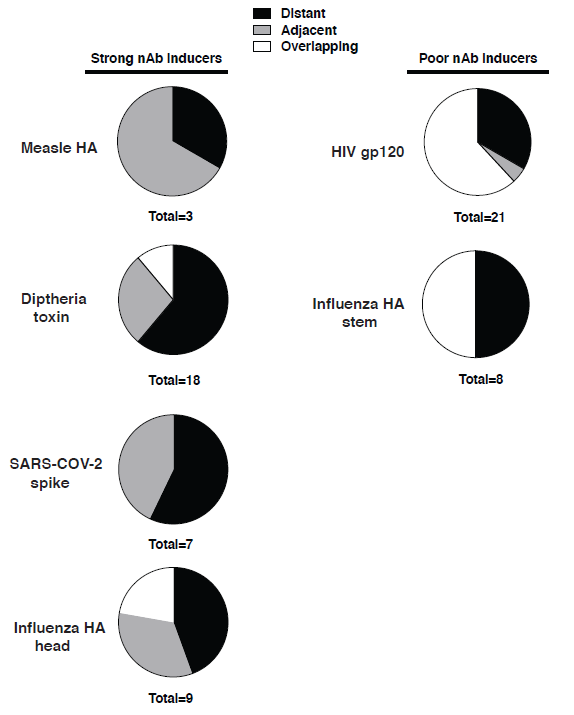


**Figure S3.** Pie charts showing percentages of distant (black), adjacent (gray), or overlapping (white) B epitopes in indicated antigens (good inducers: left; poor inducers: right). .
